# Anatomical Position of Lower Third Molar in Relation to Mandibular Canal on Cone-Beam Computed Tomography Images in A Tertiary Care Hospital: A Descriptive Cross-sectional Study

**DOI:** 10.31729/jnma.5314

**Published:** 2020-11-30

**Authors:** Bikash Chaudhary, Ujjwal Joshi, Sirjana Dahal, Alok Sagtani, Pranaya Khanal, Niroj Bhattarai

**Affiliations:** 1 Department of Oral and Maxillofacial Surgery, Kathmandu Medical College and Teaching Hospital, Bhaktapur, Nepal; 2 Department of Oral Medicine and Radiology, Kathmandu Medical College and Teaching Hospital, Bhaktapur,Nepal; 3 Department of Community and Public Health Dentistry, Kathmandu Medical College and Teaching Hospital, Bhaktapur, Nepal

**Keywords:** *anatomy*, *cone-beam computed tomography*, *inferior alveolar nerve*, *mandible*, *third molar*

## Abstract

**Introduction:**

The positional relationship between the mandibular canal with impacted mandibular third molar is the main factor of inferior alveolar nerve injury. The purpose of this study was to classify the anatomical three-dimensional relationship between the proximity of impacted mandibular third molars to the inferior alveolar canal.

**Methods:**

The descriptive cross-sectional study was conducted in the Department of Oral and Maxillofacial Surgery of a tertiary care hospital from July 2020 to August 2020 after obtaining ethical approval from the institutional review committee (Reference number 2506202001). Cone-beam computed tomography images of 200 patients' mandibular third molars were used. A convenient sampling method was used. Data were analyzed using Statistical package for the Social Sciences.

**Results:**

Mandibular canal relative to the roots of the mandibular third molar was observed on the apical side in 104 (52.0%) and 173 (86.5%) third molars had direct contact with the mandibular canal. About 36 (97.3%) lingually placed mandibular third molars had contact with the mandibular canal.

**Conclusions:**

The findings of the study conclude that most of the mandibular third molars situated lingually had a higher occurrence of mandibular nerve involvement. The anatomic structures of the mandibular third molar and the mandibular canal may be helpful to draw upon the adequate surgical plan to avoid or reduce nerve involvement.

## INTRODUCTION

The extraction of mandibular third molars is one of the most common surgical procedures done in the hospital. Ascertaining the position of impacted third molars and their contiguity with the inferior alveolar canal is of utmost importance before attempting their surgical removal to prevent the complication of an injury to inferior alveolar nerves and vessels.^1^

Previously, panoramic radiographs have been recommended as a primary radiographic investigation of choice. However, due to overlapping images on these x-ray films, it is difficult to judge the positional relationship precisely, especially in the buccolingual direction.^2–4^ With three-dimensional images, a cone-beam computed tomography (CBCT) has been widely applied in clinical work.

The study aimed to classify the three-dimensional anatomical relationship between the contiguity of impacted mandibular third molars to the inferior alveolar canal, which gives a guide to draw up the surgical plan and prevent postoperative complications.

## METHODS

A descriptive cross-sectional study was conducted in the Department of Oral and Maxillofacial Surgery, Kathmandu Medical College and Teaching Hospital, Bhaktapur, Nepal from July 2020 to August 2020. The Institutional Review Committee provided ethical clearance before conducting the study (Reference number 2506202001). CBCT imaging having the presence of unilateral or bilateral impacted mandibular third molars with ipsilateral second molars were included in the study. The images having the presence of any pathology (radiolucency that might represent a cyst, tumor, or periapical lesion), and fracture, supernumerary, or impacted teeth in the region of interest (ROI) which obscure visualization of the mandibular canal was excluded. The sample was selected by convenient sampling method. Written informed consent was obtained from all participants whose CBCT images were included in the study. The sample size has been calculated using the formula,

$$\begin{array}{ll} \text{n} & \text{= }{\text{Z}}^{\text{2}}\times \text{p}\times (\text{1}-\text{p)}/{\text{e}}^{\text{2}}\\ & \text{= }{\left(1.96\right)}^{\text{2}}\times 0.881\times 0.119/{(\text{0.05)}}^{\text{2}}\\ & \text{= }161.0998 \end{array}$$

Where,

n = required sample size
Z = 1.96 at 95% Confidence Interval
p = prevalence from previous study, 88.1%^5^
e = margin of error, 5%

Adding 20% of non-response rate, the sample size becomes 177.20978. However, a total of 200 participants were taken into the study.

The CBCT machine used in this study was Planmeca Promax 3D (Finland) and mandibular third molars were exposed at 90 kV and 12.0 mA for 14 seconds, and field of view (FOV) at 100 mm × 90 mm. Voxel size was 400 μm, and a slice thickness of axial images was 0.20 mm. Images were processed using PlanmecaRomexis software (Planmeca, Finland), to create axial, coronal, and sagittal reformatted images.

An oral and maxillofacial surgeon and oral radiologist under ideal viewing conditions interpreted all the CBCT images. The classification was based on the horizontal relationship between the mandibular third molar and the mandibular canal; the vertical relationship between the mandibular third molar and the mandibular canal and the integrity of the mandibular canal wall.^5^

**Figure 1:**
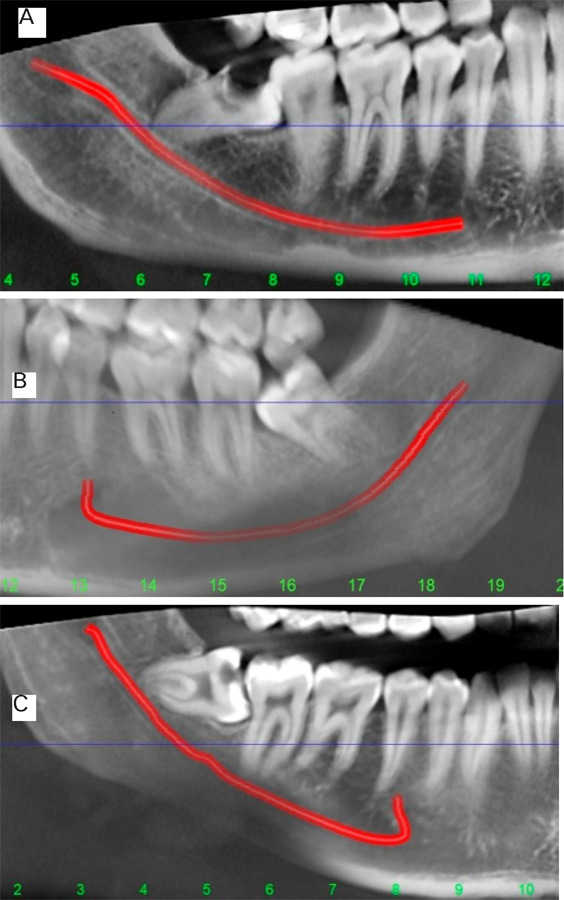
Variation in the course of mandibular canal: A) Straight projection B) Catenary-like configuration C) Progressive descent.

Interpretation of radiographs was made cautiously for the following:

The shape of the canal was observed as;

a) Straight projection: Last part of the mandibular canal was almost at the same level as a mental foramen;
b) Catenary-like configuration: Mandibular canal curled as hanging between two points;
c) Progressive descent: Descent of mandibular canal from posterior to anterior^6^ (Figure 1).

The position of the mandibular canal relative to the roots of the mandibular third molar was analyzed as:

Class I: The mandibular canal on the apical side;

Class II: The mandibular canal on the buccal side;

Class III: The mandibular canal on the lingual side;

Class IV: The mandibular canal between the roots.^5^

Based on the mandibular third molar and the mandibular canal's contact relation, the four following conditions are grouped as:

i) Mandibular third molar not in contact with the mandibular canal;
ii) Mandibular third molar does have in contact with the mandibular canal with a complete white line;
iii) Mandibular third molar does have in contact with the mandibular canal with a defective white line;
iv) Mandibular third molar penetrates the mandibular canal.^5^

Data were entered in a Microsoft Excel sheet and analyzed using Statistical Package for the Social Sciences (SPSS) software version 20. Frequency and percentage were determined for descriptive statistics.

## RESULTS

The study sample comprised of 200 patient's CBCT scans including images of 108 (54.0%) females and 92 (46.0%) males, ranging from 18 to 52 years (mean age 35 years).

The most common course of the mandibular canal observed was straight projection 123 (61.5%) followed by the catenary 56 (28.0%), and the remaining 21 (10.52%) had a progressive descent.

The mandibular canal's anatomic position concerning the mandibular third molar in the whole study population. Out of total 200 third molars, the mandibular canal concerning the mandibular third molars root was on the apical side 104 (52.0%) where as none of the third molars were observed between the roots (Table 1).

**Table 1:** Position of the mandibular canal relative to the mandibular third molar.

Position of the mandibular canal	No-Contact	Contact		
		Contact with a complete white line	Contact with a defective white line	Penetration of the mandibular canal
Class I: the mandibular canal on the apical side	14 (13)	1 (14.4)	(2.9)	20 (19.2)
Class II: the mandibular canal on the buccal side	12 (20.3)	22 (37.8)	20 (33.9)	(8)
Class III: themandibularcanal on the lingual side	1 (2.7)	0 (0.0)	14 (37.8)	22 (9)
Class IV: the mandibular canal between the roots	-	-	-	-

One hundred and seventy-three (86.5%) third molars had direct contact with the mandibular canal. Most of the individuals having mandibular canal in the lingual side had contact with the third molar 36(97.3%)(Table 2).

**Table 2:** Contact relation of the mandibular third molar and the mandibular canal in each class.

Position of Mandibular canal	No Contact n (%)	Contact n (%)
Class I	14 (13.5)	90 (86.5)
Class II	12 (20.3)	47 (79.7)
Class III	1 (2.7)	36 (97.3)

The different types of contact in Class II, and Class III. Most of the individuals with penetrating the mandibular canal 22 (81.5) had mandibular teeth located on the lingual side (Table 3).

**Table 3:** Different types of contact in Class II and Class III.

Types of contact	Class II n (%)	Class III n (%)
Contact with a complete white line	22 (100.0)	0 (0.0)
Contact with a defective white line	20 (58.8)	14 (41.2)
Penetration of the mandibular canal	5 (18.5)	22 (81.5)

## DISCUSSION

The extraction of impacted third molars is the most common procedure in the specialty of oral and maxillofacial surgery. Mandibular third molars exhibit significant differences in size, shape, and path of eruption and are also the most commonly impacted teeth. Impacted teeth could give rise to different complications like pericoronitis, dental caries, resorption, abscess formation, and cellulitis, necessitating their surgical removal. The most common complications after surgery are hemorrhage, infection, edema, trismus, alveolar osteitis, ecchymosis, and nerve damage. Among this, a serious complication of surgical extraction is an injury to inferior alveolar nerve (IAN), which accounts for 0.4 to 6 of the complications.^7–9^ Damage to the inferior alveolar nerve occurs most frequently when the mandibular third molar and nerve roots are in direct contact.^10,11^ Several clinicians have suggested a CBCT before mandibular third molar extraction could reduce the prevalence of postoperative complications. Susarla and Dodson, et al.^12^ suggested that additional information provided by CBCT images could reduce the prevalence of paraesthesia. Pre-operational analysis and evaluation help make reasonable surgical management to avoid or reduce complications.

A panoramic radiograph is done in clinical practice before extracting the mandibular third molar to evaluate the condition and risk of IAN injury.^1^ However, it can be inaccurate to evaluate the relationship in the buccolingual direction.^2–4^ Recently, CBCT provides reimaging on axial, coronal, and sagittal sections and shows the three-dimensional structures of the teeth and surrounding tissues.^3,13^ The absence of cortical integrity of the mandibular canal in CBCT images suggests direct contact of the mandibular third molar with the inferior alveolar nerve and damage or exposure of the nerve after extraction.

In this study, the most common course of the mandibular canal observed was straight projection (61.5), which is less favorable for implant placement posterior to premolars. The distribution of the other two configurations in the present study was favorable for implant placement due to mental foramen was found to be higher than the canal. This finding is in contrast to the study done by Ozturk, et al. study where the most common configuration observed was a catenary-like canal present in almost one-half of the specimens.^6^

The current study showed that 173 (86.5) third molars had a close relationship with the mandibular canal. Of these cases, a higher percentage was seen when the mandibular canal was on the lingual side. Ghaeminia, etal.^14^ demonstrated an increasing potential of IAN injury when the mandibular canal is situated lingually. Thus, we may hypothesize that the lingually positioned mandibular canal is more likely to be in contact with the mandibular third molar due to insufficient space. Though the prevalence of IAN injury is low, several operation techniques have been proposed to reduce the in risk of IAN injury.

The technique of coronectomy is one of the possible alternatives to total removal for a third mandibular molar in cases of proximity to the inferior alveolar nerve.^15^ Orthodontic extraction is another technique that decreases the risk of IAN injury by extrusion and subsequent retrieval of high-risk mandibular third molar.^16–18^ The sagittal split osteotomy was considered as one of the most common operations of mandibular deformity. It was applied to removal lower third molar close to the mandibular canal.^19,20^

This study has some limitations. This study was done considering a small sample that might not represent scans of the whole population of Kathmandu.

## CONCLUSIONS

The findings of the study conclude that most of the mandibular third molars situated lingually had a higher occurrence of mandibular nerve involvement. In this study, a three-dimensional anatomical relationship between the proximity of impacted mandibular third molars to the inferior alveolar canal and anatomic variability of the course of the mandibular canal to give guidance to draw up the surgical plan to prevent postoperative complications.
